# Supplementary material for: In Situ Growth of Nickel–Cobalt Metal Organic Frameworks Guided by a Nickel–Molybdenum Layered Double Hydroxide with Two-Dimensional Nanosheets Forming Flower-Like Struc-Tures for High-Performance Supercapacitors
Source: Nanomaterials (Basel). 2023 Jan 31;13(3):581. doi: 10.3390/nano13030581 (PMC9919709; doi:10.3390/nano13030581)
Supplement: Supplementary file 1 [file nanomaterials-13-00581-s001.zip › nanomaterials-2180856-supplementary.pdf]

nanomaterials

Supplementary Materials

In Situ Growth of Nickel–Cobalt Metal Organic Frameworks Guided by a Nickel–Molybdenum Layered Double Hydroxide with Two-Dimensional Nanosheets Forming Flower-Like Struc-Tures for High-Performance Supercapacitors

Cheng Cheng, Yongjin Zou, Fen Xu, Cuili Xiang * and Lixian Sun *

Guangxi Key Laboratory of Information Materials, Guilin University of Electronic Technology, Guilin 541004, China
* Correspondence: xiangcuili@guet.edu.cn (C.X.), sunlx@guet.edu.cn (L.S.)

1. Material characterization

The crystal structures of the samples were determined using powder X-ray diffraction (XRD; D8 ADVANCE) with Cu-Kα radiation. Scanning electron microscopy (SEM; Quanta 200) configured with energy-dispersive X-ray spectroscopy and transmission electron microscopy (TEM; JEM2010) were used to analyze the morphologies and elemental compositions of the samples. X-ray photoelectron spectroscopy (XPS; ESCA 250) measurements were obtained using an electron spectrometer with Al Kα radiation. The Brunauer–Emmett–Teller (BET; Autosorb iQ2, Quantachrome) surface area was measured using N2 adsorption–desorption isotherms at 77 K. All electrochemical measurements were performed using an electrochemical workstation (Zahner-Elektrik, Kronach).

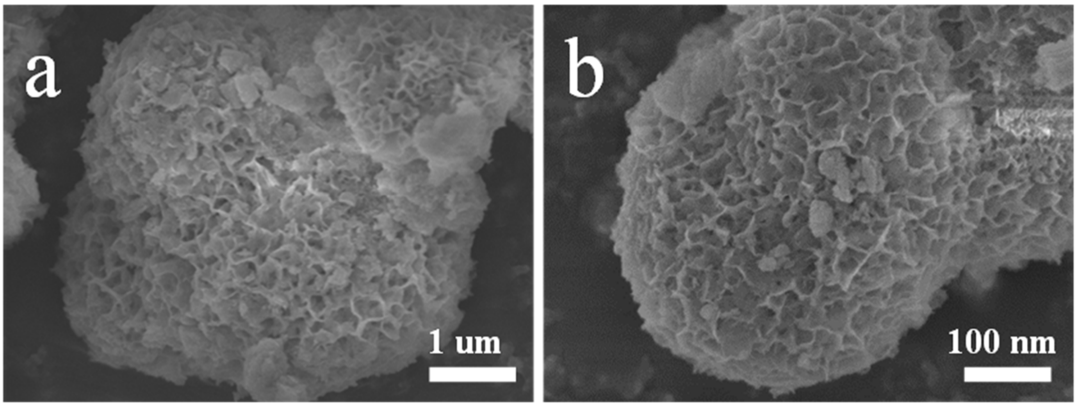

Figure S1. The appearance of NiMo-LDH@NiCo-MOF after the cycle.

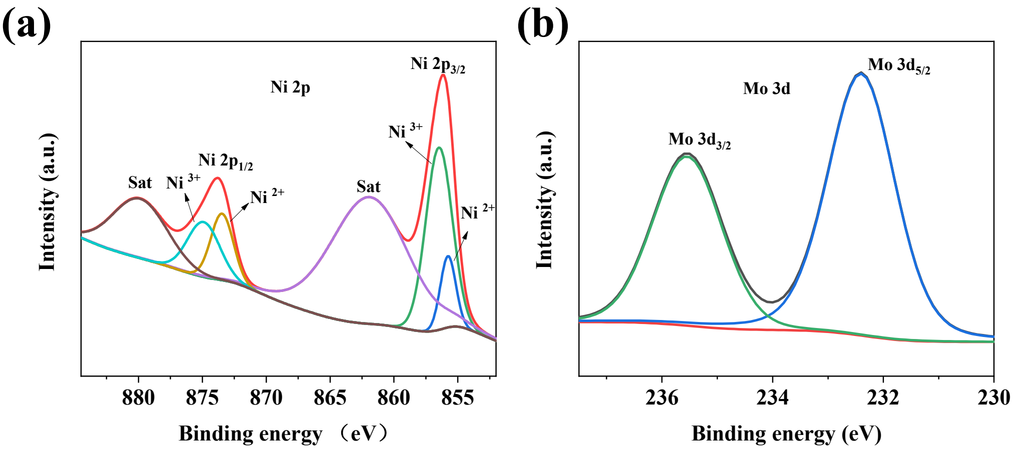

Figure S2. High-resolution XPS spectra of (**a**) Ni 2p and (**b**) Mo 3d spectra for NiMo-LDH.

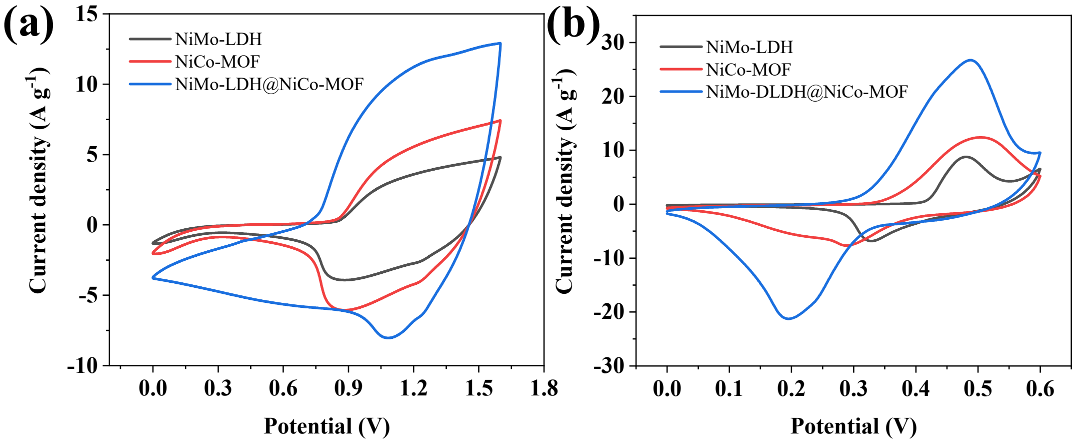

Figure S3. CV curves of NiMo-LDH, NiCo-MOF, NiMo-LDH@NiCo-MOF in two electrode (**a**) and three electrode system (**b**).

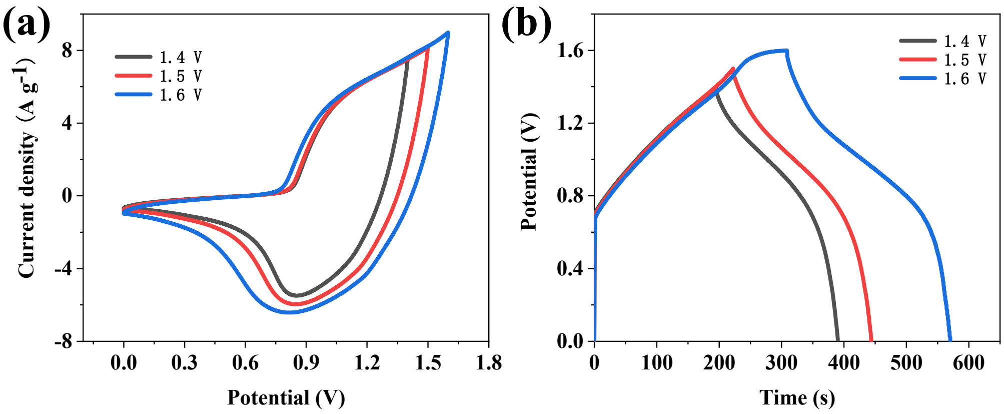

Figure S4. (**a**) CV curves for the NiMo-LDH@NiCo-MOF electrode at scan rates of 20 mV s^{-1} (**b**) GCD curves of NiMo-LDH@NiCo-MOF.
